# Expression levels of the selenium-uptake receptor LRP8, the antioxidant selenoprotein GPX1 and steroidogenic enzymes correlate in granulosa cells

**DOI:** 10.1530/RAF-23-0074

**Published:** 2024-08-02

**Authors:** Katja Hummitzsch, Jasmine E Kelly, Nicholas Hatzirodos, Wendy M Bonner, Feng Tang, Hugh H Harris, Raymond J Rodgers

**Affiliations:** 1 Robinson Research Institute, School of Biomedicine, The University of Adelaide, South Australia, Australia.; 2 Department of Chemistry, The University of Adelaide, South Australia, Australia.; 3 Adelaide Health and Medical Sciences Building, The University of Adelaide, South Australia, Australia

**Keywords:** aromatase, CYP11A1, CYP19A1, GPX1, granulosa cell, LRP8, reactive oxygen species, side-chain cleavage

## Abstract

**Graphical abstract:**

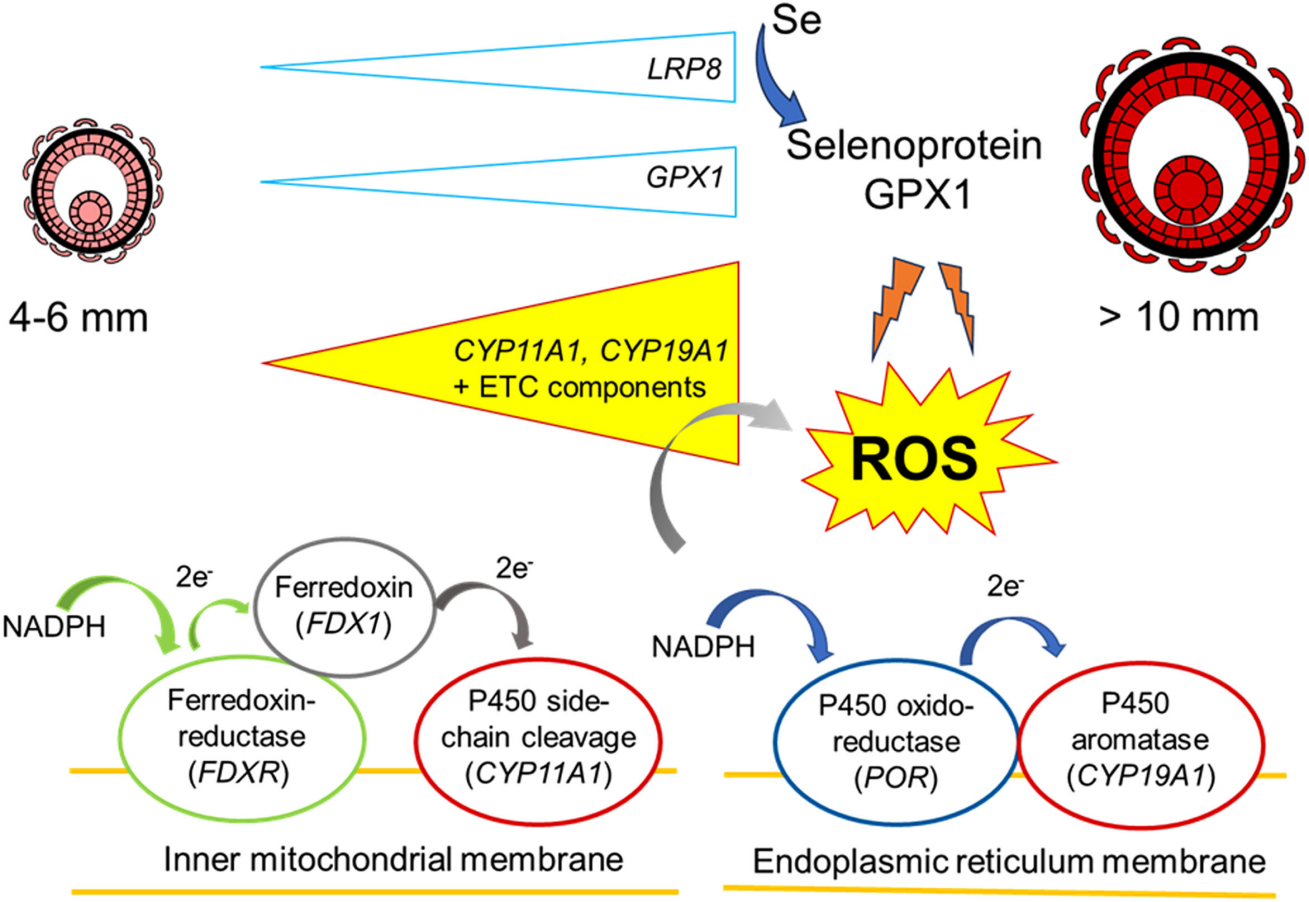

**Abstract:**

Reactive oxygen species (ROS) are a by-product of the activity of cytochrome P450 steroidogenic enzymes. Antioxidant enzymes protect against ROS damage. To identify if any particular antioxidant enzyme is used to protect against ROS produced by granulosa cells as follicles enlarge and produce oestradiol, we measured in the bovine granulosa cells the expression of two steroidogenic enzymes (*CYP11A1*, *CYP19A1*), important for progesterone and oestradiol production. We also measured the expression of the members (*FDXR*, *FDX1*, *POR*) of their electron transport chains (ETC). We measured antioxidant enzymes (*GPX*s *1*–*8*, *CAT*, *SOD*s *1* and *2*, *PRDX*s *1*–*6*, *GSR*, *TXN*, *TXNRD*s *1*–*3*). Since selenium is an active component of GPXs, the selenium-uptake receptors (*LRP*s *2* and *8*) were measured. Only the selenium-dependent *GPX1* showed the same increase in expression as the steroidogenic enzymes did with increasing follicle size. *GPX4* and* PRDX2/6* decreased with follicle size, whereas *SOD1/2*,* CAT*, *GSR*, and* TXNRD3* were lowest at the intermediate sizes. The other antioxidant enzymes were unchanged or expressed at low levels. The expression of the selenium-uptake receptor *LRP8* also increased significantly with follicle size. Correlation analysis revealed statistically significant and strongly positive correlations of the steroidogenic enzymes and their ETCs with both *GPX1* and *LRP8*. These results demonstrate a relationship between the expression of genes involved in steroidogenesis and selenium-containing antioxidant defence mechanisms. They suggest that during the late stages of folliculogenesis, granulosa cells are dependent on sufficient expression of *GPX1* and the selenium transporter *LRP8* to counteract increasing ROS levels caused by the production of steroid hormones.

**Lay summary:**

In the ovary, eggs are housed in follicles which contain the cells that produce oestrogen in the days leading up to ovulation of the egg. Oestrogen is produced by the action of enzymes. However, some of these enzymes also produce by-products called reactive oxygen species (ROS). These are harmful to eggs. Fortunately, cells have protective antioxidant enzymes that can neutralise ROS. This study was interested in which particular antioxidant enzyme(s) might be involved in neutralising the ROS in follicle cells. It was found that only one antioxidant enzyme, GPX1, appeared to be co-regulated with the enzymes that produce oestrogen and progesterone in the follicular cells. GPX1 contains the essential mineral selenium. In summary, this study has identified which antioxidant appears to be involved in neutralising ROS in the days leading to ovulation. It highlights the importance of selenium in the diet.

## Introduction

The ovary has two major functions: first, the production and release of mature oocytes, capable of being fertilised, and secondly, the biosynthesis of steroid hormones, including oestradiol and progesterone. Steroid hormone production depends on the expression and activity of cytochrome P450 enzymes. Cytochrome P450 cholesterol side-chain cleavage (P450scc; *CYP11A1* gene) and P450 17α-hydroxylase (P450c17; *CYP17A1* gene) are involved in converting cholesterol into androstenedione in the follicular thecal cells. The latter is then converted via multiple steps into oestrogen by P450 aromatase (P450arom; *CYP19A1* gene) in follicular granulosa cells. Cytochrome P450 enzymes rely on electron transport chains (ETCs) to gain electrons to produce oxygen radicals in order to hydroxylate their substrates. Critically though, cytochrome P450 enzymes leak electrons to O_2_, thereby producing reactive oxygen species (ROS). These include superoxide radicals and peroxides, hydroxyl radicals, and singlet oxygen (Hanukoglu *et al.* 1993). High levels of ROS in the face of inadequate levels of antioxidants (ROS scavengers) lead to oxidative stress which results in cellular damage due to the oxidation of proteins, lipids, and DNA (Sies 2000). This represents a risk to the developing oocyte because ROS can cause deterioration of cohesion of sister chromatids, allowing aneuploidy to occur (Perkins *et al.* 2016). However, cells can protect against ROS damage with molecular free-radical scavengers, such as β-carotene, α-tocopherol, ascorbate, glutathione, or thioredoxin and through the activity of antioxidant enzymes (e.g. superoxide dismutases (SOD), catalase (CAT), and glutathione peroxidases (GPX)). The active site of any of these enzymes contains metal/metalloids such as zinc, copper, or selenium.

We previously identified a selenium-containing enzyme, glutathione peroxidase 1 (GPX1; (Ceko *et al.* 2015*b*)), to be important in ovarian follicles during the follicular phase, when increasing production of oestradiol and progesterone occurs. Using synchrotron X-ray fluorescence imaging to study trace element distributions in the ovary, we found within a narrow window of time that selenium, in particular, accumulated in the granulosa cells in large pre-ovulatory follicles in cows (Ceko *et al.* 2015*b*). Using our RNA microarray data (granulosa cells, thecal cells, stroma, and cumulus–oocyte complexes), we examined members of the family of selenoproteins and through further detailed analyses by RNA and protein quantitation identified that only glutathione peroxidase 1 (GPX1) could account for the observed accumulation of selenium in granulosa cells particularly in larger antral follicles (Ceko *et al.* 2015*b*). Taken together, these observations are consistent with the expression of GPX1 in granulosa cells being indicative of a need for antioxidant activity in the late follicular phase of the oestrous cycle.

To date, a comprehensive analysis of all antioxidant enzymes in granulosa cells during folliculogenesis has not been completed. Therefore, we isolated granulosa cells from small, intermediate, and large bovine antral follicles and examined the mRNA expression of members of the major classes of antioxidant enzymes including glutathione peroxidase, superoxide dismutase, catalase, glutathione-disulfide reductase (GSR), peroxiredoxins (PRDX), thioredoxins (TXN), and thioredoxin reductases (TXNRD). We also examined the selenium-uptake receptors, LDL receptor related protein 2 (LRP2/megalin) and LRP8 (also ApoER2). To gain insight into which, if any, antioxidant genes might be specifically regulated to protect against ROS due to steroidogenesis during folliculogenesis, we conducted correlation analyses between the expression of antioxidant genes with that of *CYP11A1*, *CYP19A1* and their ETC members – P450 oxidoreductase (gene *POR*) for P450arom and ferredoxin reductase (gene *FDXR*) and ferredoxin (gene *FDX1*) for P450scc.

## Materials and methods

### Granulosa cell isolation

Bovine ovaries from heifers were collected at an abattoir (Thomas Foods International, Murray Bridge, SA, Australia) and washed once in 70% ethanol and twice with Hank’s Balanced Salt Solution (HBSS) without Ca^2+^/Mg^2+^ (Sigma-Aldrich). Small (4–6 mm diameter), intermediate (7–10 mm diameter), and large (> 10 mm diameter) antral follicles were dissected from the ovaries. Each follicle was transferred individually into a sterile petri dish containing 10 mL HBSS without Ca^2+^/Mg^2+^ (intermediate and large follicles) or 10 ml HBSS with Ca^2+^/Mg^2+^ with 50 µg/mL DNase I (small follicles) and cut open with sterile scissors. Only follicles that exhibited clear follicular fluid, and additionally the large follicles that had visible vasculature, were used. A blunt-ended glass pipette was used to scrape the inside of the follicle and the inside was flushed with HBSS. Cell suspensions were centrifuged for 5 min at 483 ***g*** and the supernatant discarded. Red blood cells were lysed using 500 µL Red Blood Cell Lysis Buffer (8.3 g/L ammonium chloride in 0.01M Tris-HCl buffer, pH 7.5 +/− 0.2; Sigma-Aldrich) for 30 s and then pelleted as described above. Pellets were frozen at −80°C for RNA extraction.

### RNA isolation and quantitative real-time PCR

RNA was extracted from the granulosa cells by using TRIzol^™^ reagent, following the manufacturer’s instructions (Life Technologies/Thermo Fisher Scientific Australia Pty. Ltd), and resuspended in 20 µL of nuclease-free H_2_O. Ten micrograms or less of each sample was treated with 2 U of DNase I for 20 min at 37°C and the enzyme was removed using a DNase inactivation reagent (Thermo Fisher Scientific). Two hundred nanograms of DNase-treated RNA were used for reverse transcription reactions with or without Superscript RT III (Thermo Fisher Scientific) to generate cDNA or a negative control to detect genomic DNA contamination, respectively.

Quantitative real-time PCR of the cell samples for the target genes and the housekeeping genes *GAPDH* and* RPL19* was performed using a Rotor-Gene 6000 series 1.7 thermal cycler (Corbett Life Science, Concord, NSW, Australia). cDNA dilutions were amplified in 10 µL reactions containing 5 µL of Power SYBR™ Green PCR Master Mix (Applied Biosystems/Life Technologies), 0.2 µL each of reverse and forward primers (Sigma Aldrich; Table 1), and 2 µL of the 1/10 cDNA dilution and 2.6 µL DEPC-treated water. PCR amplification of the cDNA samples was carried out in duplicate at 95°C for 15 s, followed by 60°C for 60 s for a total of 40 cycles. The Rotor-Gene 6000 software (Q Series, Qiagen) was used to determine the cycle threshold (Ct) values at a threshold of 0.05 normalised fluorescence units. Gene expression was determined by the mean of 2^−∆Ct^, where ∆Ct represents the target gene Ct – (mean of *GAPDH* and *RPL19*) Ct.

**Table 1:** Primers used for qRT-PCR.

Gene	Forward (5′–3′)	Reverse (5′–3′)	Product size (bp)
*GAPDH*	ACCACTTTGGCATCGTGGAG	GGGCCATCCACAGTCTTCTG	76
*RPL19*	GATCCGGAAGCTGATCAAAG	TACCCATATGCCTGCCTTTC	113
*FDXR*	TGGCCTTCACCATAAAGGAG	TCCTGGAGACCCAAGAAATC	91
*FDX1*	AACAGATAGATCGCGGTTGG	ACGGCATCAGGTACTCGAAC	81
*CYP11A1*	CACTTTCGCCACATCGAGAA	TGAATGATATAAACTGACTCCAAATTGC	86
*POR*	ACGGACGTGATCCTGTTTTC	TCGTGGTCTGAATCTTGGTG	106
*CYP19A1*	GGCTATGTGGACGTGTTGACC	TGAGAAGGAGAGCTTGCCATG	142
*GPX1*	CATCGCTCTGAGGCACAACGGT	TGCCCAAACTGGTTGCAGGGGA	112
*GPX2*	CCAACTCAACGAGCTGCAATG	GGACGTACTTGAGGCTGTTC	125
*GPX3*	CATCCTGCCTTCTCTCCCTG	GAGGGCCCCGTACTCATAGA	107
*GPX4*	ACCCTCTGTGGAAATGGATG	CAGCCGTTCTTGTCAATGAG	103
*GPX5*	GTTGGGATTTCCCTGTAACC	CACCATTCACATCCCCTTTC	104
*GPX6*	TATGGAAGCCCTCACCCTCAA	GGGAAAGCCCAGCACAACTA	174
*GPX7*	TACAGCGCTTGTGAGGAAAC	TCTGCTTTGGTCACTCATGC	108
*GPX8*	TTGGCTTTTCCATGCAATCAGTT	TCCAAAAGTTCCACCTTGGTT	193
*LRP2*	GCCAGCAAGGAACCAAATAG	AGCAAGGGTTGTTGTTGACC	133
*LRP8*	CTGTCATTGGGATCATCGTG	TCTTCCGCTTCCAGTTTCTC	87
*CAT*	TTGTCTGCAAGGGAGAAAGC	TGCAGGAGAATCTTCCATCC	133
*SOD1*	GGTGGTCCATGAAAAACCAG	ATTACACCACAGGCCAAACG	96
*SOD2*	CGTGACTTTGGTTCCTTTGC	CTTATTGAAGCCGAGCCAAC	96
*PRDX1*	TGGTGCTTCTGTGGATTCTC	AATGTTCATGGGTCCCAGTC	85
*PRDX2*	TGCCTTCAAGGAGGTGAAAC	TGGGGCACACAAAGGTAAAG	89
*PRDX3*	AACACACCGAGGAAGAATGG	CTCCGTTGGGGTCAATTATG	148
*PRDX4*	GCACCTTATTGGGAAGGAAC	GGCGATGATTTCAGTTGGAC	138
*PRDX5*	AATCCTCGAGTGGACGTTTG	GGCCCCTTTTCAAATACCTC	114
*PRDX6*	CACTGGCAGGAACTTTGATG	TTCCTCTTCAGGGATGGTTG	136
*GSR*	GCCTAGGAATAACCAGTGATGG	AGCACCAACAATGACACTGC	71
*TXN*	GGTGGGTGAATTTTCTGGAG	CAATGGCTGGTCATGTCTTC	95
*TXNRD1*	TCCCCGGTGACAAAGAATAC	ACCAGCAAGAAATCCAGCAC	125
*TXNRD2*	AGTTCCAGAAACCGCAAGTC	TCACCGATGGCATAGATGTG	123
*TXNRD3*	TGAGCACCACAAAGTCAAGG	GATGACAAACTTCGCAGCAG	73

### Statistical analysis

Differences between small, intermediate, and large antral follicle groups were examined using IBM SPSS, version 28. Two-way ANOVA and *post hoc* Tukey’s test were used to detect statistically significant differences of *P* < 0.05 between the three groups of follicle sizes. Pearson’s correlation coefficients were calculated using the data from all follicles, and graphs were plotted using GraphPad Prism 6 v008.

## Results

### Expression of steroidogenic enzymes and their electron transport chain partners

**Figure 1:**
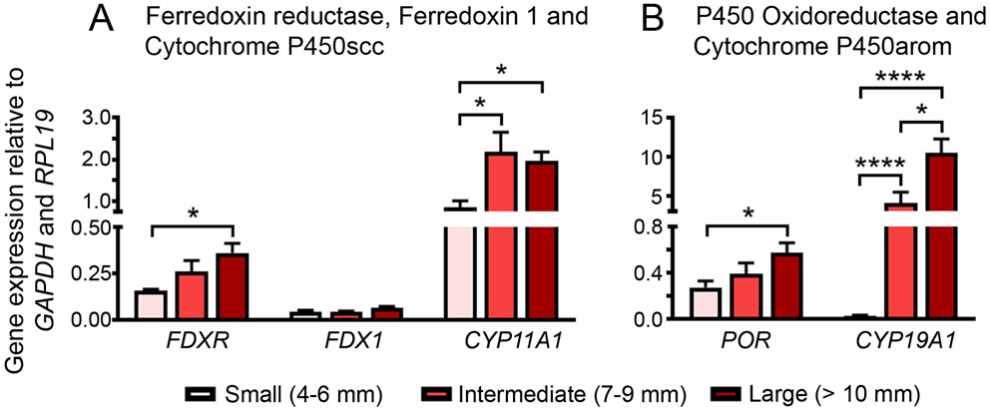
Gene expression analysis of steroidogenic enzymes during follicle growth. mRNA expression of (A) cytochrome P450 side chain cleavage enzyme (*CYP11A1*) and its electron transport chain partners ferredoxin reductase (*FDXR*) and ferredoxin (*FDX1*), and (B) cytochrome P450 aromatase (*CYP19A1*) and its electron transport partner P450 oxidoreductase (*POR*) in small (4–6 mm, *n* = 6), intermediate (7–10 mm, *n* = 6) and large (> 10 mm, *n* = 6) antral follicles. Two-way ANOVA and *post hoc* Tukey’s test were used to identify statistical significance between groups; *P* values: *< 0.05, ****< 0.0001.

The gene expression of *CYP11A1* and *CYP19A1* was significantly increased in bovine granulosa cells in intermediate and large antral follicles compared to those from small antral follicles (Fig. 1A and B). Their corresponding reductases, *FDXR* and *POR,* showed a significant increase between small and large antral follicles (Fig. 1A and B). Gene expression of *FDX1* was independent of follicular size and levels were relatively low (Fig. 1A). In the large follicles* CYP19A1* was more highly expressed than *CYP11A1* (Fig 1).

### Expression of anti-oxidative defence systems

**Figure 2:**
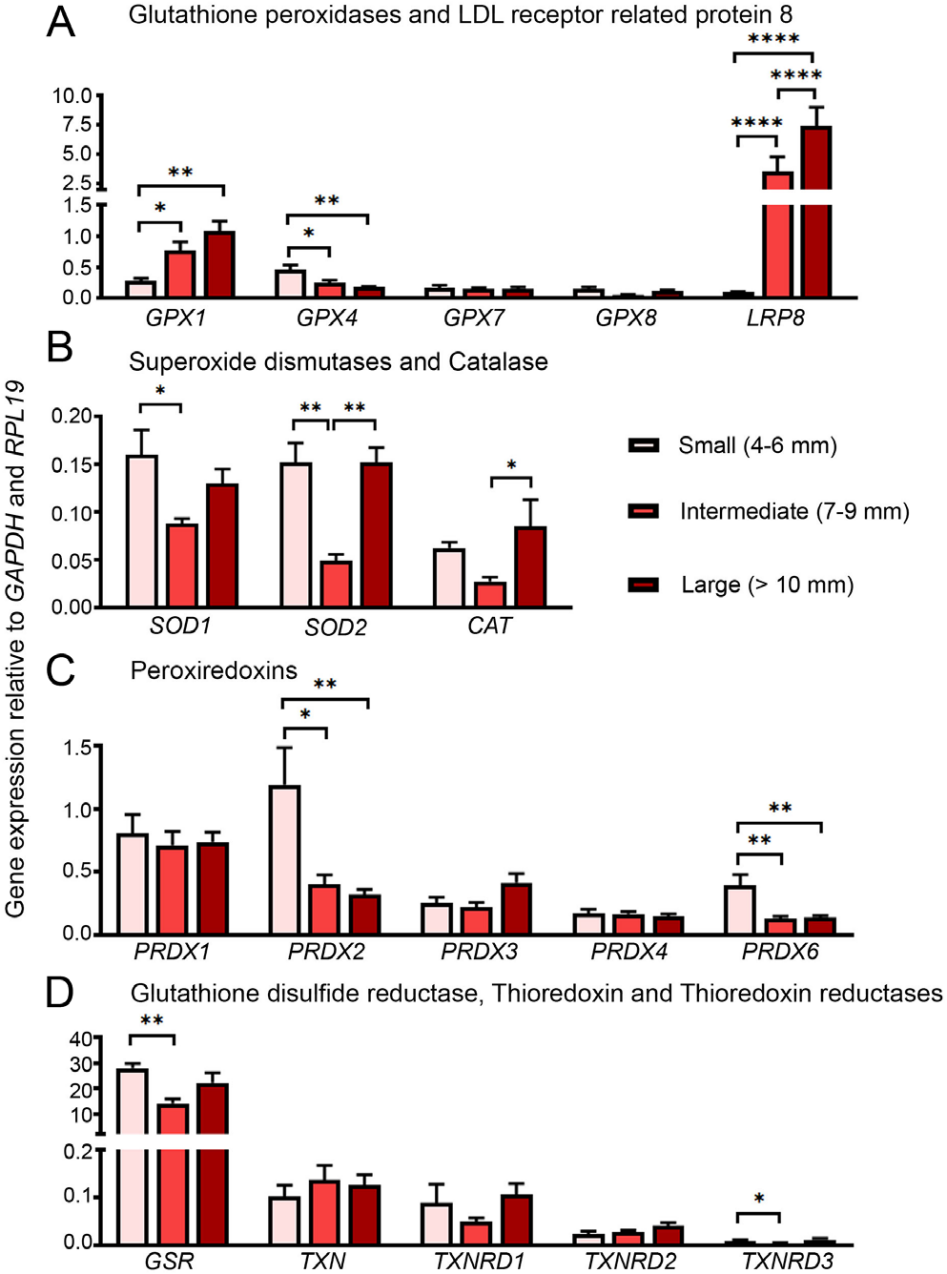
Gene expression analysis of antioxidant enzymes during follicle growth. mRNA expression of (A) glutathione peroxidases and selenium-uptake receptor *LRP8*, (B) superoxide dismutases and catalase, (C) peroxiredoxins, (D) glutathione disulfide reductase, thioredoxin, and thioredoxin reductases in small (4–6 mm, *n* = 3–6), intermediate (7–10 mm, *n* = 5–6), and large (> 10 mm, *n* = 4–6) antral follicles. Two-way ANOVA and *post hoc* Tukey’s test was used to identify statistical significance between groups; *P* values: *< 0.05, **< 0.01, ****< 0.0001.

Of all antioxidant genes analysed (Fig. 2), only *GPX1* expression was significantly increased in intermediate and large antral follicles compared to small antral follicles (Fig. 2A). *GPX4*, on the other hand, was most highly expressed in small antral follicles compared with intermediate and large antral follicles (Fig. 2A). *GPX7* was expressed independently of follicular size (Fig. 2A). Expression levels for *GPX2*, *GPX3*, *GPX5*, and *GPX6* are not presented as graphs here as their expression was very low or nil in bovine granulosa cells of all follicular sizes (Supplementary Table 1, see section on supplementary materials given at the end of this article). The gene expression of the selenium uptake-receptor *LRP8* followed the same expression pattern as *GPX1* (Fig. 2A) and the steroidogenic enzymes (Fig. 1), whereas *LRP2* was expressed at only very low levels in bovine granulosa cells of all follicular sizes (Supplementary Table 1).

Both *SOD1* and *SOD2* were significantly decreased at the intermediate follicular size compared to small and large follicles (Fig. 2B). A similar, but not significant, pattern could be observed for *CAT* (Fig. 2B). Gene expression analysis of the peroxiredoxin family (Fig. 2C) revealed that *PRDX2* and *PRDX6* were highest expressed in small antral follicles (Fig. 2A). *PRDX1*, *PRDX3*, and *PRDX4* were expressed independently of follicular size (Fig. 2C) and *PRDX5* was very lowly expressed in bovine granulosa cells of all follicular sizes analysed (Supplementary Table 1).

*GSR* was the highest expressed antioxidant analysed (Fig. 2). It was expressed at significantly higher levels in small antral follicles compared to intermediate and large antral follicles (Fig. 2D). *TXN* and the thioredoxin reductases *TXNRD1* and *TXNRD2* were expressed independently of follicular size, whereas *TXNRD3* showed the same expression pattern as *GSR,* being expressed at the highest levels in small antral follicles (Fig. 2D).

### Correlation analysis between steroidogenesis and anti-oxidative defence systems

**Figure 3:**
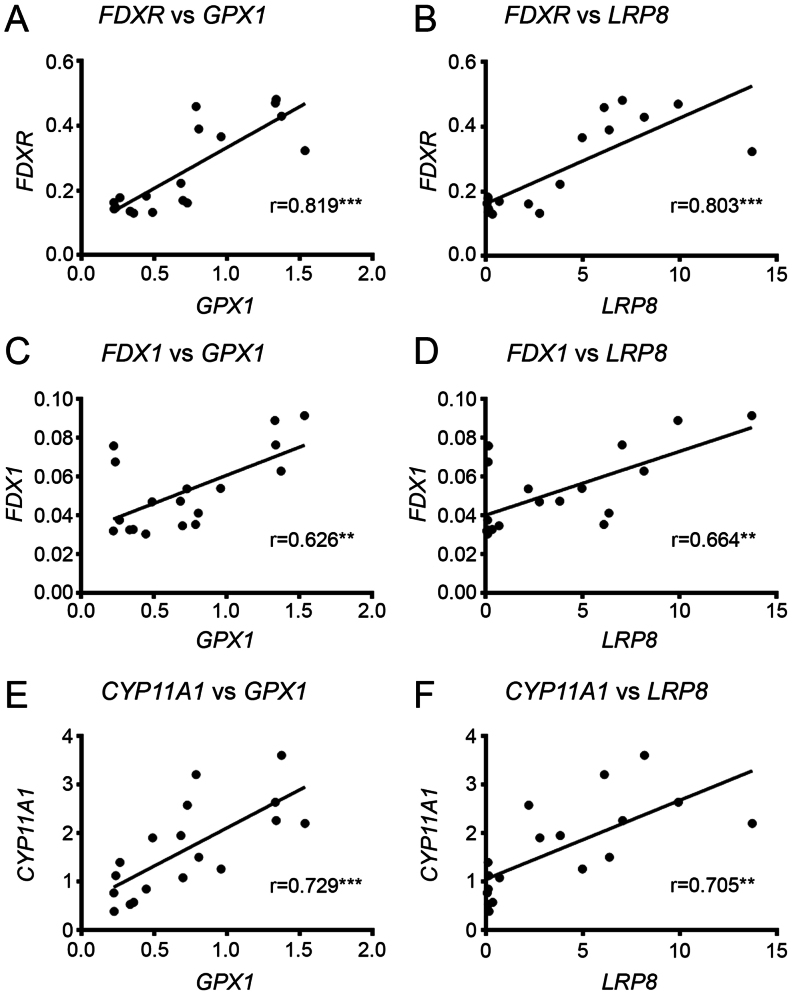
Correlation of *CYP11A1* and its electron transport chain members with *GPX1* and *LRP8*. Scatter plot of normalised expression of *FDXR* with (A) *GPX1* and (B) *LRP8*. Scatter plot of normalised expression of *FDX* with (C) *GPX1* and (D) *LRP8*. Scatter plot of normalised expression of *CYP11A1* with (E) *GPX1* and (F) *LRP8*. Pearson’s correlation coefficient is shown in each graph with *P* values: **< 0.01, ***< 0.001.

**Figure 4:**
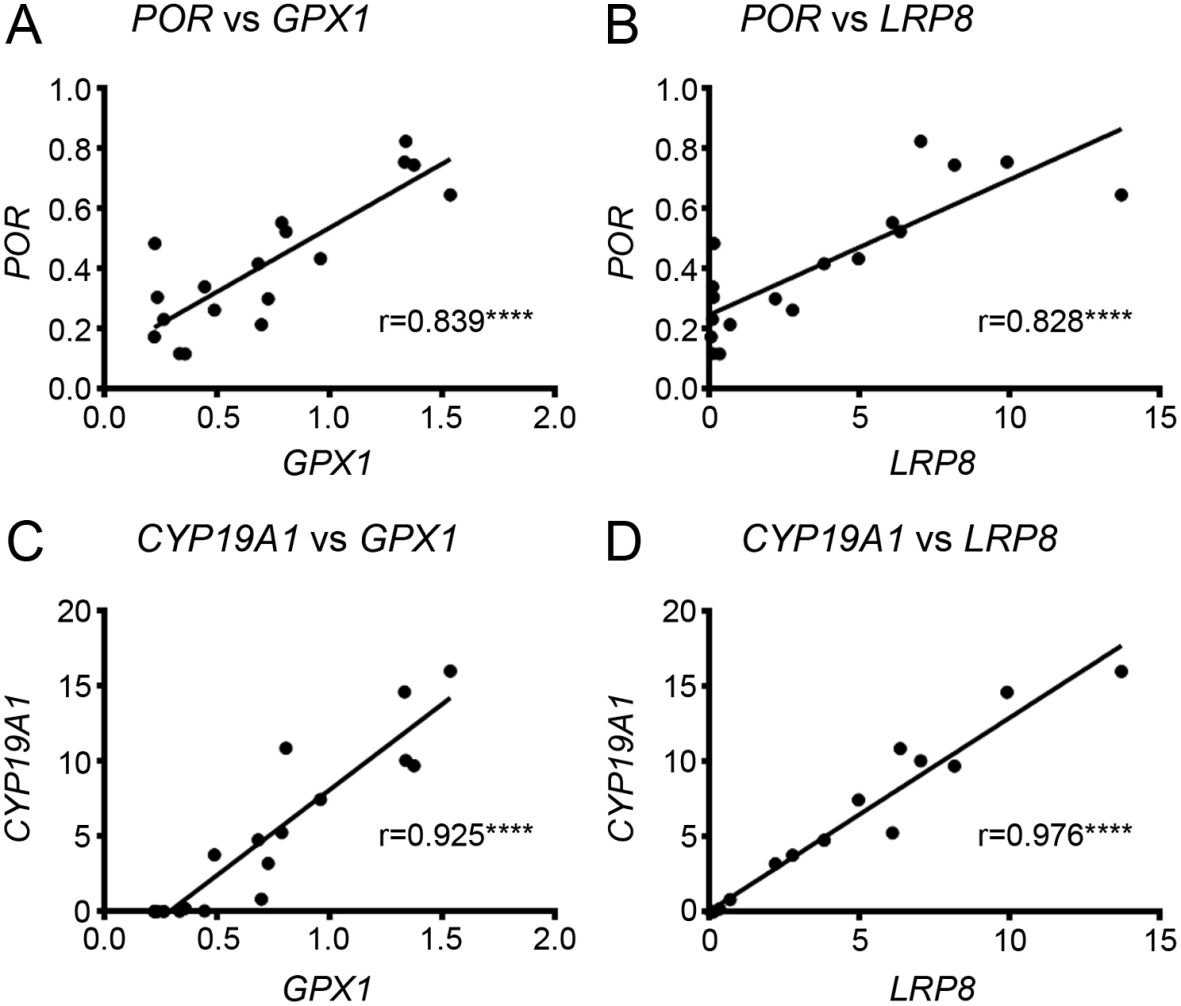
Correlation of *CYP19A1* and its electron transport chain partner *POR* with *GPX1* and *LRP8*. Scatter plot of normalised expression of *POR* with (A) *GPX1* and (B) *LRP8*. Scatter plot of normalised expression of *FDX* with (C) *GPX1* and (D) *LRP8*. Pearson’s correlation coefficient is shown in each graph with *P* values: ****< 0.0001.

**Figure 5:**
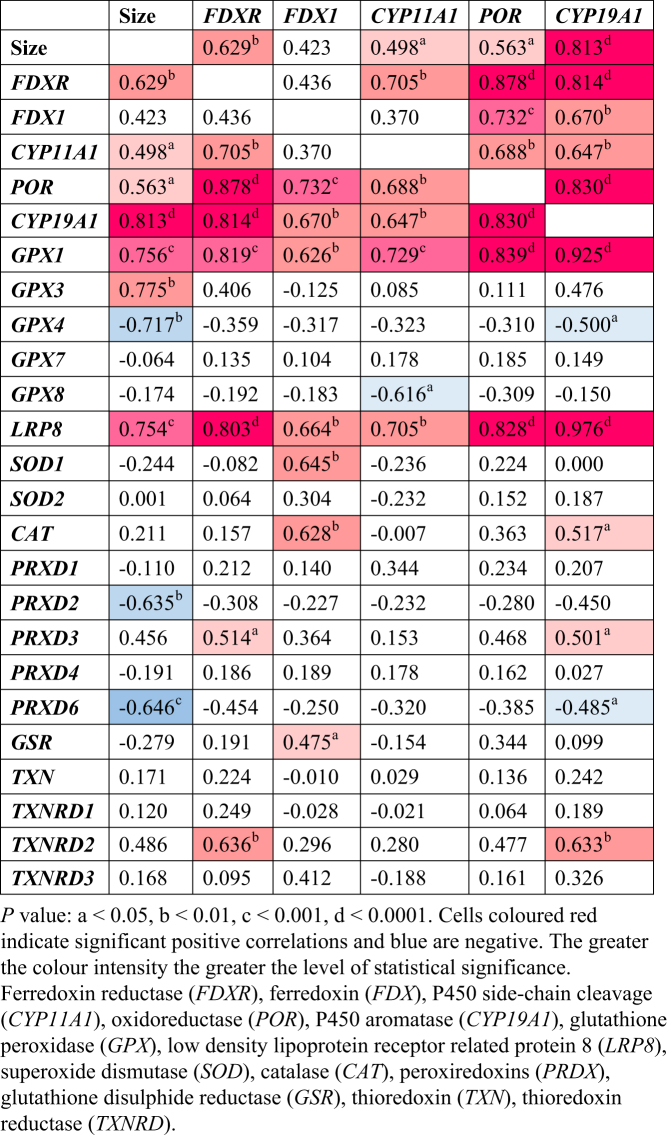
Pearson’s correlation coefficients for steroidogenic genes with size, and genes involved in ovarian steroidogenesis and antioxidant response (*r* values).

**Figure 6:**
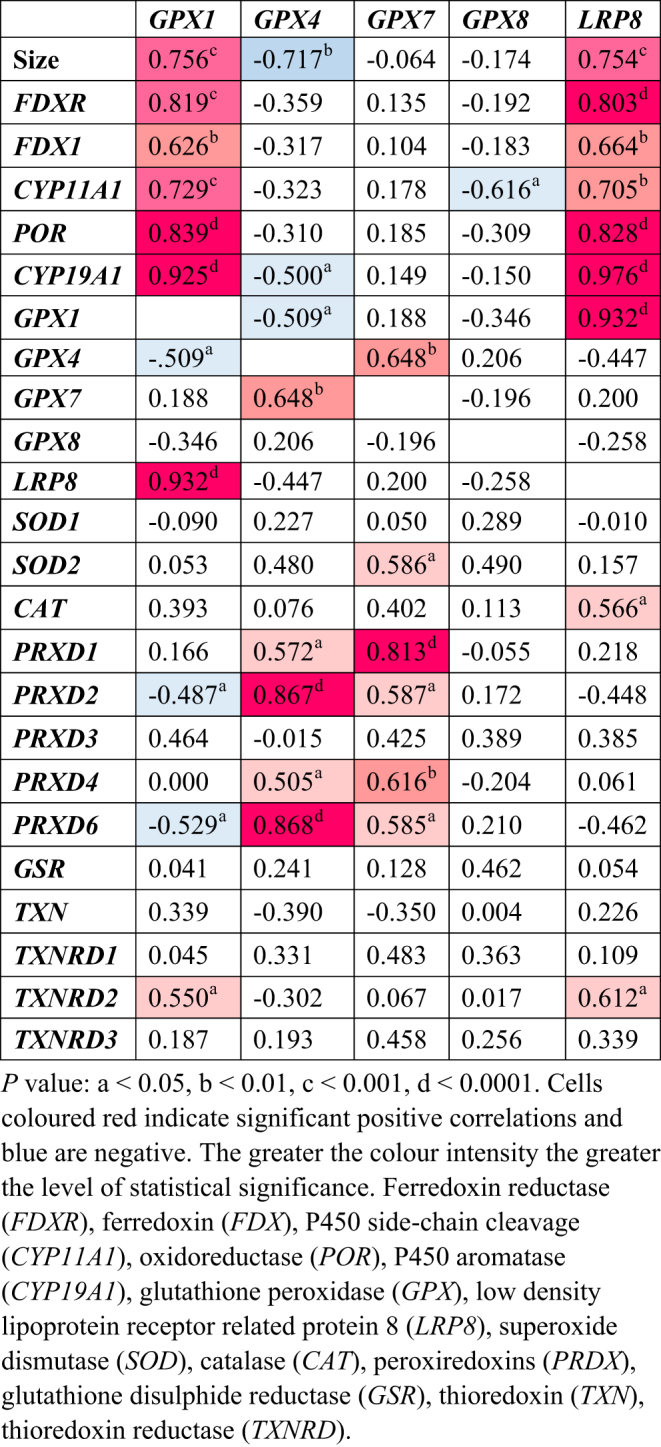
Pearson’s correlation coefficients for glutathione peroxidases and selenium-uptake receptor *LRP8* with size, and genes involved in ovarian steroidogenesis and antioxidant response (*r* values).

*FDXR*,* FDX1*, and* CYP11A1* were each strongly positively correlated with *GPX1* and *LRP8* (Fig. 3). The positive correlations between *POR* and *CYP19A1*, and the anti-oxidative defence system *GPX1* and *LRP8* were even stronger (Fig. 4). Of the remaining antioxidant genes analysed, all relationships to the steroidogenic genes were very weakly associated in either a positive or negative manner (Figs. 5 and 6). There was a positive correlation between *FDX1* and *SOD1* (Fig. 5), as well as *CAT* and *GSR* (Fig. 5), and between *CYP19A1* and *CAT* (Fig. 5). *GPX4* was negatively correlated with *CYP19A1* (Fig. 5).

**Figure 7:**
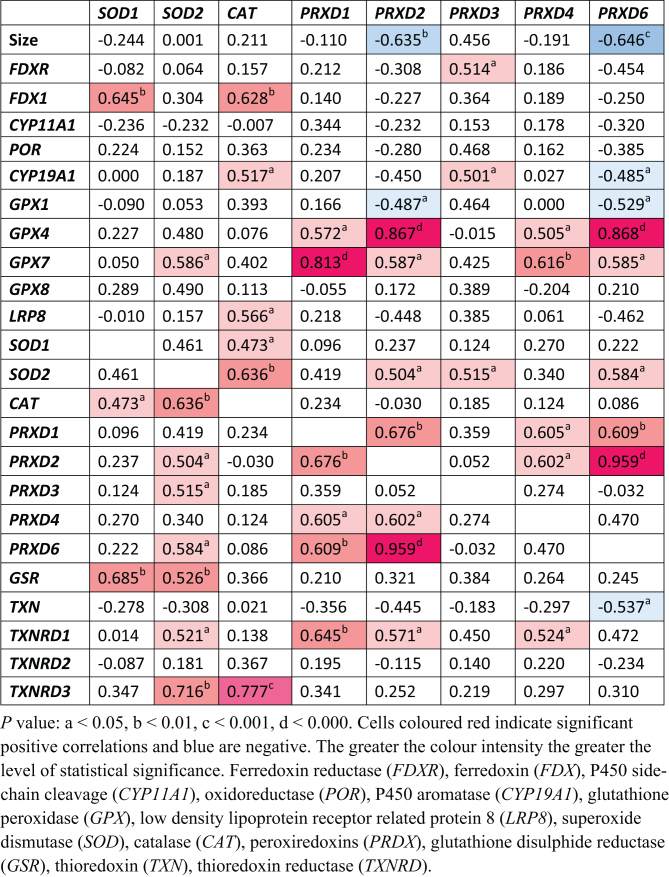
Pearson’s correlation coefficients for superoxide dismutases (*SOD*), catalase (*CAT*), and peroxiredoxins (*PRDX1-6*) with size, and genes involved in ovarian steroidogenesis and antioxidant response (*r* values).

**Figure 8:**
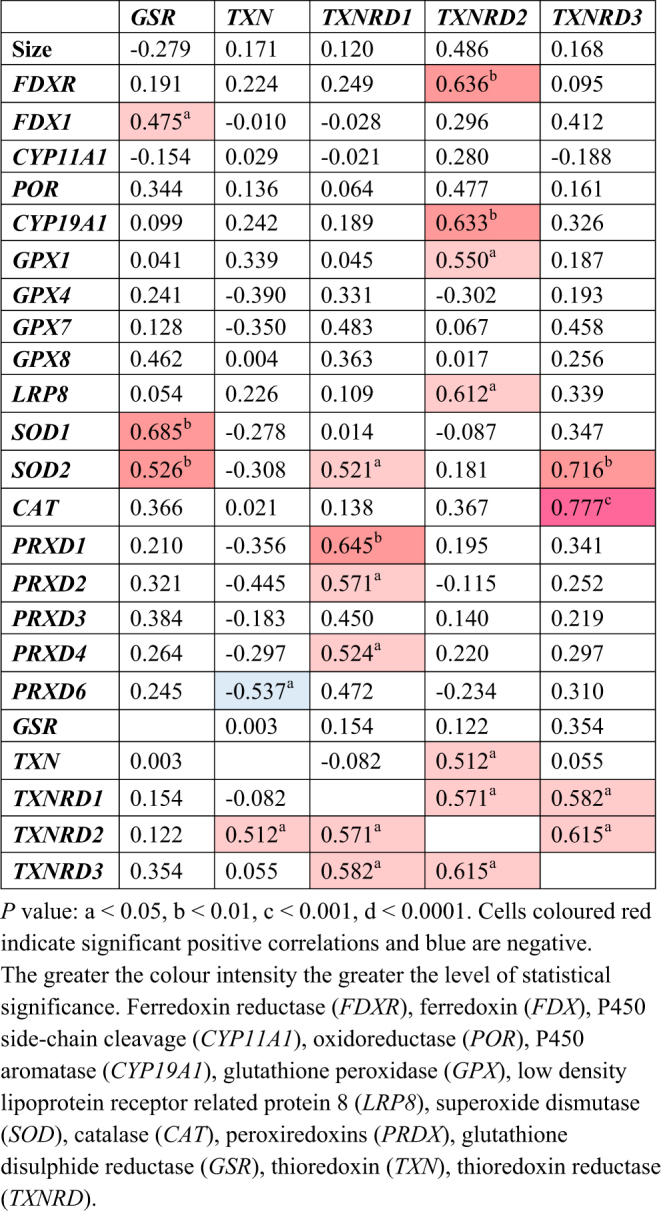
Pearson’s correlation coefficients for glutathione disulphide reductase (*GSR*), thioredoxin (*TXN*), and thioredoxin reductases (*TXNRD1-3*) with size, and genes involved in ovarian steroidogenesis and antioxidant response (*r* values).

Aside from correlations between steroidogenesis and antioxidants, there were some correlations between different antioxidant systems. *GPX4* positively correlated with *GPX7*, *SOD2*, and *PRDX1*, *2*, *4*, and *6*, but was negatively correlated with *GPX1* and *LRP8* (Fig. 6). *GPX7* was also positively correlated with *PRDX1*, *2*, *4*, and *6* (Fig. 6). Both superoxide dismutases, *SOD1* and *SOD2*, were positively correlated with *CAT* and *GSR* (Fig. 7). *SOD2* was additionally positively correlated with *PRDX2* and *PRDX6*, and negatively correlated with *TXN* (Fig. 7). *PRDX1* positively correlated with *PRDX2*, *PRDX4*, and *PRDX6*, as well as *TXNRD1* (Fig. 7). The same correlation pattern was seen with *PRDX2* (Fig. 6). *PRDX4* also positively correlated with *PRDX1* and *PRDX2* and *TXNRD1* (Fig. 7). *PRDX6* positively correlated with *PRDX1* and *PRDX2*, and negatively with *TXN* (Fig. 7). *TXN* was positively correlated with *TXNRD2*, which was also negatively correlated with *GSR* (Fig. 8). Lastly, *TXNRD1* was positively correlated with the other two members, *TXNRD2* and *TXNRD3* (Fig. 8).

## Discussion

This study measured the expression of antioxidant genes in bovine granulosa cells during the later stages of follicular growth when the expression of steroidogenic cytochrome P450 enzymes increases. We examined the relationships in expression between the steroidogenic genes and antioxidant genes in maturing granulosa cells. We identified one antioxidant enzyme, a selenoprotein, and a selenium-uptake receptor whose expression levels tracked and correlated highly with expression of steroidogenic enzymes, suggesting that they might be important for protection against ROS produced by steroidogenesis in follicles in their later stages of growth.

Cytochrome P450s are a large group of enzymes, and many are expressed in the liver where they conduct the biotransformation of drugs and other exogenous chemical compounds. These liver P450 enzymes are microsomal. Only six P450 enzymes are involved in steroidogenesis, three of them are microsomal, and three are mitochondrial (Hanukoglu *et al.* 1993). Mitochondrial cytochrome P450s are thought to have arisen from a common microsomal P450 ancestor and adapted to the existing ETC in mitochondria, which differs from that in the endoplasmic reticulum (Hartz *et al.* 2021). Transfer of electrons via ETCs to P450s, in particular in mitochondria, can be quite inefficient (Hanukoglu 2006). One study found that only 85% of electrons were transferred from NADPH to the mitochondrial CYP11A1 during hydroxylation of cholesterol (Rapoport *et al.* 1995). Hence, 15% of electrons leaked. This leakage of electrons leads to the production of ROS (Hanukoglu 2006).

Since steroidogenesis is a source of ROS, steroidogenic cells must protect against ROS by way of either antioxidant enzymes or non-enzymatic antioxidants, or both. Not all steroidogenic cells are the same in the amount of steroid or type of steroid hormone they produce, in the cytochrome P450s they express, and in the proportions of microsomal or mitochondrial steroidogenesis. Hence, one could expect differences between different steroidogenic cells in how they handle ROS produced by steroidogenesis. Additionally, access to antioxidants may affect which ROS detoxification approaches are utilised by different steroidogenic cells. For example, the corpus luteum and adrenal cortex are both very steroidogenic compared with granulosa cells. The corpus luteum is highly vascularised and has, therefore, access to antioxidants via the bloodstream and is rich in ascorbate (vitamin C; (Young *et al.* 1995, Kodaman *et al.* 1998)), alpha-tocopherol (vitamin E; (Schweigert 2003)) and beta-carotene (Young *et al.* 1995, Haliloglu *et al.* 2002); the latter giving the corpus luteum its characteristic yellow colour. All three non-enzymatic ROS scavengers have their highest levels when the corpus luteum is most steroidogenically active. The adrenal cortex, another highly vascularised tissue, has high levels of ascorbate (Hornig 1975), alpha-tocopherol, and retinol (Azhar *et al.* 1995). In contrast to the corpus luteum and the adrenal cortex, which are both well vascularised, the granulosa cell layers of the follicle are avascular and granulosa cells are far less steroidogenic than luteal or adrenal cells. Hence, the expression of antioxidant enzymes is likely key for defence against ROS in granulosa cells.

In the current study, we sought to identify which antioxidant enzymes might be deployed to defend against the ROS produced as a consequence of steroidogenesis in granulosa cells. We examined the expression of all antioxidant enzymes at different stages of granulosa cell maturation when steroidogenesis increases. We found striking relationships in the levels of expression of *GPX1* and both *CYP11A1* and *CYP19A1* and members of their ETCs. Since GPXs contain selenium, we examined the two selenium-uptake receptors and found that expression of *LRP8* was also highly correlated with expression of both *CYP11A1* and *CYP19A1*and their ETCs. This increase in *GPX1* and *LRP8* expression is also in agreement with increasing concentrations of selenium in maturing granulosa cells as observed previously (Ceko *et al.* 2015*a*,*b*). There appeared to be no such relationship to steroidogenesis with any of the other antioxidant enzymes examined.

The uptake and accumulation of selenium in granulosa cells in maturing follicles is not just important for the catalytic activity of GPXs. Selenium also regulates the transcript abundance and translational efficiency of *GPX*s (Weiss Sachdev & Sunde 2001). This occurs because the codon for selenocysteine is UGA (Low & Berry 1996), which acts as a stop codon in the absence or deficiency of selenocysteine (Low & Berry 1996). When selenium is deficient, degradation of the truncated GPXs occurs (Weiss Sachdev & Sunde 2001). Selenium deficiency has been shown to cause ovarian degeneration and follicular atresia in rats (Grabek *et al.* 1991) and has been related to miscarriages (Barrington *et al.* 1996). During selenium deficiency, the activity of liver GPX1 in rats drops to 3% of its normal activity (Weiss Sachdev & Sunde 2001), whereas the activity of GPX4 is better maintained. This is due to GPX1’s lower ranking in the selenoprotein hierarchy, which causes a faster cut-off from selenium sources than for other selenoproteins (Burk & Hill 2015). Selenium supplementation reduced ROS-induced oxidative stress and increased GPX1 activity in preantral follicles (Abedelahi *et al.* 2008, Abedelahi *et al.* 2010) and increased the blastocyst rate in mice (Yang *et al.* 2019). In an *in vitro* study using bovine granulosa cells from small (< 5 mm) and large (> 8 mm) antral follicles, selenium supplementation significantly increased proliferation of the granulosa cells from the small follicles, and oestrogen production by granulosa cells from both groups (Basini & Tamanini 2000). Interestingly, selenium was able to significantly reduce nitric oxide production in the granulosa cells of small and large antral follicles, confirming a role in ROS scavenging (Basini & Tamanini 2000).

The importance of a defence mechanism against ROS is illustrated by what damage ROS can do in follicles. Increased ROS levels can cause granulosa cells to undergo apoptosis (Yang *et al.* 2017). Women with impaired fertility have increased ROS levels in their granulosa cells, and lower fertilisation and embryo development rates (Karuputhula *et al.* 2013, Lai *et al.* 2018) suggesting that ROS production in granulosa cells affects oocyte quality. Evidence shows that oxidative stress in oocytes leads to premature loss of cohesions and errors in chromosome segregation and hence aneuploidy, an abnormal number of chromosomes (Perkins *et al.* 2016, Mihalas *et al*. 2017). The oocyte is more prone to aneuploidy compared with somatic cells because of its spindle assembly checkpoint being less stringent with abnormal chromosome behaviour (Nagaoka *et al.* 2011). Increased expression of SOD1/2 successfully suppressed segregation errors (Perkin *et al*. 2019 ), and supplementation of mice with different antioxidants increased IVF outcomes and embryo development (Truong & Gardner 2017). To protect the oocyte against increasing ROS levels during folliculogenesis due to granulosa cell steroidogenesis requires the granulosa cells to increase their total antioxidative capacity adequately to diminish ROS.

The importance of a defence mechanism against ROS is additionally illustrated by the steroidogenic behaviour of granulosa cells in response to either high ROS or inadequate ROS defences. Hydrogen peroxide inhibited LH- or cAMP-stimulated progesterone production by rat granulosa cells (Margolin *et al.* 1990) and luteal cells (Musicki *et al.* 1994). It inhibited progesterone and oestrogen synthesis in human luteal cells (Endo *et al.* 1993). Superoxide (Gatzuli *et al.* 1991) and lipid hydroperoxide (Kodaman *et al.* 1994) also inhibited stimulated steroidogenesis in rat luteal cells. Mice deficient in *Sod2* expression have reduced ovarian steroidogenesis due to reduced cholesterol transport into the mitochondria and downregulation of *Star, Cyp11a1, Cyp17a1* and *Cyp19a1* (Zaidi *et al.* 2021). These findings suggest that in the face of high ROS levels, steroidogenic cells have the ability to reduce or block stimulation of steroidogenesis and thereby avoid additional oxidative stress.

## Conclusion

The findings are the first to demonstrate a relationship between the expression of genes involved in steroidogenesis and selenium-containing antioxidant defence. There were no strong relationships between follicular size, steroidogenesis, and antioxidants for any of the antioxidant enzymes aside from *GPX1*. Why GPX1 is the only antioxidant significantly upregulated during follicular development is yet to be determined. The present data, however, provide further insight into how selenium could be beneficial for reproductive health and function. Considering that ROS can be produced during cytochrome P450 reactions, and steroidogenic granulosa cells are in close proximity to the oocyte, a selenium and GPX1-led defence in granulosa cells may be important for protecting the oocyte and reducing the incidence of aneuploidy.

## Supplementary Materials

Table S1. Expression values relative to housekeeping genes GAPDH and RPL19 (2<sup>-ΔCt</sup>) of extremely low or nil expressed antioxidant enzymes.

## Declaration of interest

The authors report that there is no conflict of interest that could be perceived as prejudicing the impartiality of the study reported.

## Funding

This work was supported by National Health and Medical Research Councilhttp://dx.doi.org/10.13039/501100000265 of Australia (GTN1143289). KH was supported by The University of Adelaidehttp://dx.doi.org/10.13039/501100001786’s Robinson Research Institute Career Development Fellowship and a Building on Ideas Grant. FT was supported by Adelaide University (https://www.adelaide.edu.au/) China Fee Scholarship and the Faculty of Sciences, Engineering and Technology, University of Adelaidehttp://dx.doi.org/10.13039/501100001786.

## Author contribution statement

Study design: KH, JRK, HHH, RJR; tissue collection: NH, WMB; data collection: KH, JRK, NH, WMB; data analysis: KH, JRK, HHH, RJR; manuscript writing: KH, JRK, FT, HHH, RJR; manuscript review and approval: all authors.
