# Supplementary material for: Expression levels of the selenium-uptake receptor LRP8, the antioxidant selenoprotein GPX1 and steroidogenic enzymes correlate in granulosa cells
Source: Reprod Fertil. 2024 Aug 2;5(3):e230074. doi: 10.1530/RAF-23-0074 (PMC11301534; doi:10.1530/RAF-23-0074)
Supplement: Table S1. Expression values relative to housekeeping genes GAPDH and RPL19 (2-ΔCt) of extremely low or nil expressed antioxidant enzymes. [file supplementary_table_s1.pdf]

Table S1. Expression values relative to housekeeping genes *GAPDH* and *RPL19* ($2^{-\Delta Ct}$) of extremely low or nil expressed antioxidant enzymes.

Follicle size		*GPX2*	*GPX3*	*GPX5*	*GPX6*	*LRP2*	*PRDX5*
small	1	0.00014	0.00087	0.00077	0.03921	0.00022	0.00264
	2	0.00011	0.00064	0.00104	0.02210	0.00006	0.00001
	3	0.00025	0.00038	0.00236	0.00334	0.00015	0.00081
	4	-	-	0.00065	-	0.00004	0.00274
	5	-	-	0.00092	-	0.00007	0.00007
	6	-	-	0.00007	-	-	0.00006
intermediate	1	0.00121	0.00259	0.00012	0.00338	0.0003	0.00080
	2	0.00030	0.00048	0.00001	0.00000	0.00062	0.00082
	3	0.00098	0.00233	0.00001	0.00000	0.00020	0.00216
	4	0.00260	0.00117	0.00009	0.00000	-	0.00131
	5	0.00081	0.00073	0.00039	0.00005	-	0.00058
	6	-	-	0.00031	-	-	-
large	1	0.00065	0.00416	0.00028	0.00000	0.00128	0.00080
	2	0.00141	0.00052	0.00000	0.00002	0.00033	0.00032
	3	0.00248	0.00427	0.00005	0.00000	0.00016	0.00071
	4	0.00047	0.00439	0.00048	0.00006	-	0.00065
	5	-	-	0.00027	-	-	0.00083
	6	-	-	0.00029	-	-	0.00036

- Not enough cDNA left for this sample to run qPCR
